# Era of the 4D animal model

**DOI:** 10.1002/ame2.12306

**Published:** 2023-02-27

**Authors:** Lihong Zhang, Jindan Guo, Jiangning Liu

**Affiliations:** ^1^ NHC Key Laboratory of Human Disease Comparative Medicine, Beijing Key Laboratory for Animal Models of Emerging and Remerging Infectious Diseases Institute of Laboratory Animal Science, Chinese Academy of Medical Sciences and Comparative Medicine Center, Peking Union Medical College Beijing China

**Keywords:** animal model, database, digitally display, human diseases, spatiotemporal landscapes

## Abstract

Revealing the entire dynamics of pathogenesis is critical for understanding, preventing and treating human disease but is limited by systematic clinical sampling. This drawback can be overcome with animal model studies. Recent advances in phenotyping, omics and bioinformatics technologies promote the development of the 4D animal model to simulate and digitally display the spatiotemporal landscapes of phenotypes and molecular dynamics in human diseases and reveal novel targets for diagnosis and therapy. In this commentary, the origin, supporting technologies, content, function and application, and advantages of 4D animal models over clinical studies and traditional animal models, as well as their limitations, are presented.

## Graphical abstract

A 4D database for human disease was established based on the 3‐dimensional (3D) spatial resolution phenotypic information of disease and then integrating the fourth dimension (time resolution) by comparing the 3D phenotypic data at different key timepoints, such as disease onset, development and recovery, which together constitute the spatiotemporal phenotype of a disease.

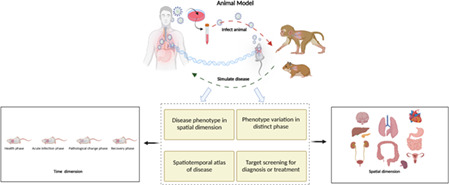

The onset of disease symptoms is driven by a series of pathological changes in the body that are caused by interior or exterior etiological agents and manifest as specific disease‐related physical outcomes and behaviors. Revealing the entire dynamics of in vivo pathogenesis, which could be described as uncovering the black box of disease in the body, is the basis for the prevention, diagnosis and treatment of disease.

## 1 LIMITATION OF CLINICAL STUDIES

In clinical studies, the comparison of genomes from different patients with those of population controls is used to elucidate the genetic mechanisms of disease, and transcriptome‐wide association analysis and colocalization are applied to infer the effect of gene expression on disease occurrence and severity.^1^ Additionally, proteomic techniques targeting the expression of proteins can be used to investigate the cellular composition and spatial landscape of tissue pathology at single‐cell resolution.^2^ Moreover, advances in imaging technology have enabled the integration of time‐resolution data and phenotypic 3‐dimensional image information to provide a 4‐dimensional (4D) image atlas at high resolution.^3^

Well‐established databases of human diseases, such as MalaCards, provide an integrated compendium of annotated disease data for disease representation and scrutiny.^4^ Nevertheless, comprehensive sampling of internal organs from a sufficient number of patients following the time dimension of disease progression is usually not feasible. In addition, mapping the spatiotemporal landscape of disease in the clinic needs to exclude the interference of variance in genetic background, age, host microbiome and fundamental disease characteristics of the patient population, which will frequently affect the calculation of corresponding values as well as the continuity of the data curve along the timeline of disease progression.^5^, ^6^

## 2 WHAT IS A 4D ANIMAL MODEL?

**FIGURE 1:**
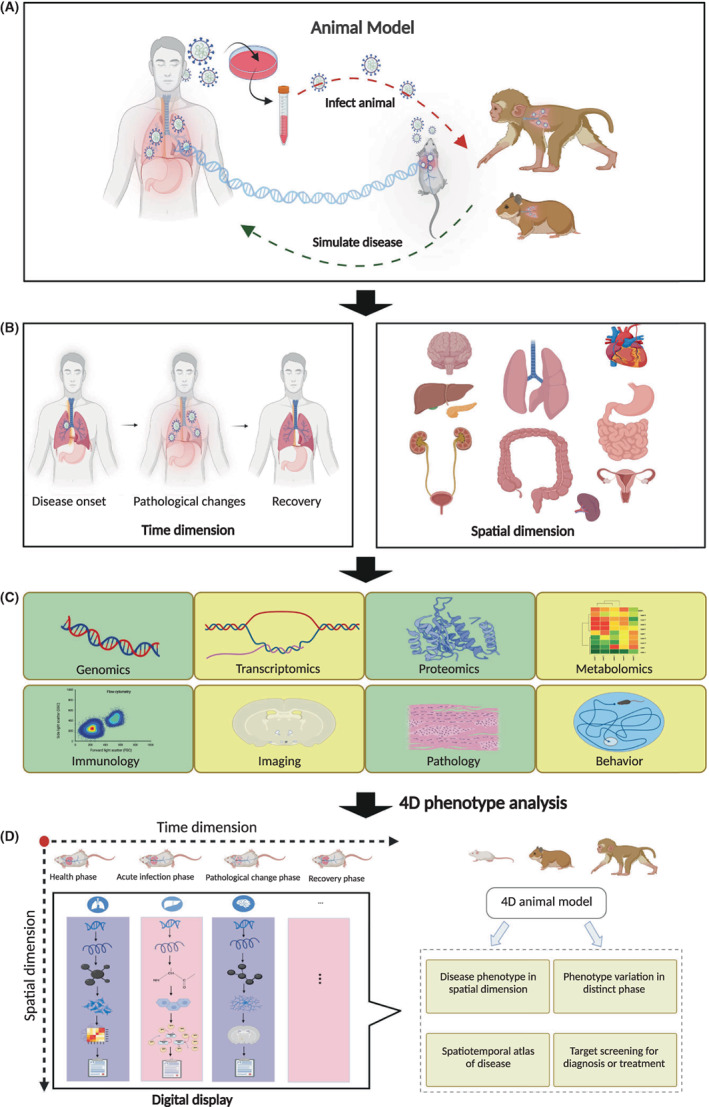
Outline of the development of the 4D animal model. (A) Selection of the ideal animal model based on the similarity of pathogenesis and phenotypes between different animal models and human disease. (B) Design of the phenotypic flow in time and spatial dimensions according to the characteristics of the disease. (C) Spatiotemporal screening of the disease landscape of phenotypes and molecular dynamics. (D) Analysis and digital display of the database of the 4D animal model with the choice of time and spatial dimensions reflecting the characteristics of the target disease.

To better reveal the spatiotemporal landscapes of human diseases and support the studies on pathogenesis and developments on drug candidates, we proposed the concept of the ‘4D animal model’. A 4D animal model is conceived as a digital platform based on a 4D database consisting of information on human disease based on an ideal animal model. A 4D animal model is generated by integrating the 3‐dimensional (3D) phenotypic information of a disease with the 4th dimension (time resolution) information by comparing the 3D phenotypic data at different key time points, such as disease onset, development and recovery, which together constitute the spatiotemporal phenotype of a disease. The phenotypes cover the in vivo clinical symptoms, such as the main changes in organs, tissues or cells related to pathogenesis, including but not limited to those included in atlases on genomics, epigenetics, transcriptomics, proteomics, metabolomics, physiology, biochemistry, immunology, pathology, imaging, behaviors and clinical symptoms. After comprehensive data are collected and summarized, representing the total information on the disease, a 4D animal model digital platform can be established to provide a public website with corresponding software for use by scientific communities The following is an example of a potential 4D animal model of an infectious disease (Figure 1).

1. For a certain time point covered by the database of the 4D animal model, for example, at 5 days post infection, with the targeted pathogen such as a virus, the phenotypic landscape, including virology, immunology, pathology, imaging, symptoms and molecular dynamics, can be demonstrated upon request.^7^
2. Any tissue or organ site of interest can be chosen, e.g., the left upper lobe of the lung, and users can then retrieve and compare the local information of phenotypes such as viral replication, tissue‐resident immune response, pathological injury, imaging and molecular dynamics between different time points.^8^
3. The spatiotemporal atlas is mapped for a given phenotype. Taking tissue‐resident memory T (T_RM_) cells as an example, the 4D animal model can clarify the anatomical distribution of functional subpopulations of T_RM_ cells in peripheral blood, lymph nodes in different parts of the body, immune organs, pathogen‐targeted organs and other organs, together with the tendency towards variation in the function, frequency and fate of the T_RM_ cells following the host immune response stimulated upon infection, in a similar but more comprehensive way than is possible in the clinic.^9^
4. Focusing on a targeted gene related to infection or pathogenesis, a 4D animal model can combine genomics, epigenetics, transcriptomics and proteomics data at the single‐cell level to exhibit the expression of genes and then compare the profile of the gene product to control animals in space and time dimensions. This will facilitate clarification of the function and correlation of each gene involved in the pathogenesis of disease as well as prediction of the precise location and time that the gene plays its functional role.^10^, ^11^

## 3 DEVELOPMENT OF THE 4D ANIMAL MODEL

Animal models simulating human disease bridge the gaps in deep tissue sampling at different time points in clinical studies and have evolved to be the most ideal tool for unbiased and undisturbed understanding of the internal dynamics of diseases. Previous long‐term animal studies, especially studies of mice and monkeys, have systematically developed phenotyping techniques for animal models of human diseases, which cover the physiology, biochemistry, immunology, pathology, imaging, behaviors and clinical symptom phenotypes associated with disease. Omics technologies are constantly improving and developing rapidly; for example, single‐nucleus RNA sequencing (snRNA‐seq) and single‐cell RNA sequencing (scRNA‐seq) have been used to establish a large‐scale cell transcriptomic atlas that encompasses over 1 million cells across 45 tissues of the nonhuman primate (NHP) *Macaca fascicularis*.^11^ Currently, next‐generation sequencing and imaging‐based approaches have together established spatial transcriptomics techniques to measure the expression levels of all or most genes systematically throughout the tissue space and have been adopted to study development and disease pathogenesis.^12^ Furthermore, high spatial resolution omics sequencing combining DNA nanoball (DNB)‐patterned arrays and in situ RNA capture has been used to generate a mouse organogenesis spatiotemporal transcriptomic atlas.^10^ The above‐mentioned technologies, together with advancements in epigenomic, proteomic, metabolomic and immunomic techniques, have facilitated the dynamic mapping of molecules with single‐cell resolution and high sensitivity in spatiotemporal dimensions during animal development and disease. With these technologies, several databases of mouse models for human diseases have been developed, such as the MGI resource, which includes multiple databases or resources, such as the Mouse Genome Database, Gene Expression Database, Mouse Tumor Biology Database and Gene Ontology.^13^ However, data integration and format standardization are the main barriers to data utilization, and a 4D animal model with 3‐dimensional (3D) phenotypic information of a disease over a systematic time course remains to be established.

## 4 ADVANTAGES OF A 4D ANIMAL MODEL

Assuming that the above functional aim is achieved, the application prospects and advantages of a 4D animal model are expected to be important and extensive, especially in the following areas. First, we can systematically understand the nature of human disease based on the whole spatiotemporal landscape of molecular dynamics and phenotypes.^11^ Second, we can screen a series of molecular targets related to early warning, pathogenesis, host immunity and repair during disease progression, which will provide comprehensive information on target selection for the development of diagnostic reagents, target drugs and drug combinations against multiple targets.^7^ Third, comprehensive and standardized data will contribute to guiding the development and precise application of animal models to research on pathogenesis and vaccines as well as drug evaluation.^14^ Fourth, repeated use of animal models for a specific human disease can fulfill the demands of a tremendous number of projects that overlap to a great extent in research content, which will prevent the unnecessary use of animals and improve animal welfare.^13^ Finally, based on a 4D animal model, multiple data streams related to, for example, drug treatment, gene regulation and other interventions regarding phenotypes and molecular dynamics, will be integrated gradually, and then a 4D+ digital and artificial intelligent animal model can be generated via deep learning and applied to predict the effect of drug candidates.^15^

## 5 FACTORS INFLUENCING THE DESIGN AND DEVELOPMENT OF A 4D ANIMAL MODEL

Providing a 4D animal model is critical for future advances in medicine; however, its development requires significant funding, including funds for animal's purchase, phenotyping, omics analysis, bioinformatics calculations and website establishment. Therefore, each 4D animal model for a specific human disease should be carefully designed. Most importantly, it is essential to consider the similarity between the selected animal model and the corresponding human disease. Due to the genetic differences between humans and animals, as well as the diversity in microbiota, nutrition and other elements, animal models usually fail to simulate the natural state or full clinical manifestation of disease.^14^, ^16^, ^17^ Misuse of animal models that are inconsistent with the key clinical factors related to pathogenesis or phenotypes associated with research and drug targets will lead to lower reproducibility of animal experimental results in clinical trials.^15^, ^18^, ^19^ In addition to the simulation issues, it is equally important that 4D animal models are shared to avoid repeated construction and allow availability of complete phenotypic information. To achieve complete phenotyping of animal models, it is essential that research tools for the chosen animal model are available. For example, hamsters are susceptible to SARS‐CoV‐2 infection and are suitable for demonstrating the pathogenesis of COVID‐19,^20^ but specific antibodies against hamsters are currently limited compared to the research tools available for studying the immune response of mice or monkeys to infection. This represents a major barrier to completing the immunology component of a 4D animal model.^21^ Furthermore, to meet the minimum requirements for use, the space‐dimensional phenotypes should include the targeted organ and related immune or circulatory system and contain the main phenotypes for critical time points of disease progression. Regarding resolution, while spatial transcriptomics in the targeted organs is not accessible, techniques such as transcriptomics and proteomics should be applied to monitor the molecular dynamics of genes in addition to common phenotypes such as those related to etiology, physiology, immunology, pathology, imaging, behavior and symptoms.^2^ Finally, to facilitate calculations or analyses based on data of interest in the 4D animal model by individual scientists, rather than mere demonstration of the results, all raw data and images should be shared online, and consistency in the genetic and microbial background of selected animals, as well as in the methods, reagents and machines used in animal model development and phenotyping, needs to be considered to help with statistical analysis of the data.^4^

## 6 PERSPECTIVES ON THE 4D ANIMAL MODEL

With the development of phenotyping, omics and bioinformatics technologies, the 4D animal model is expected to comprehensively demonstrate the entire dynamic landscape of human diseases in spatiotemporal dimensions. The 4D animal model based on standardized disease data will be shared by the scientific community and will act as a ‘black box’ of human diseases, predict target molecules for treatment and prevention, guide animal model selection and development, and reduce the numbers of animals used in experiments. To achieve this goal, several 4D animal models for human diseases should be developed as first attempts, and then any disadvantages of these models should be determined and the models should subsequently be optimized.

## AUTHOR CONTRIBUTIONS

Conceptualization: Jiangning Liu; Methodology: Lihong Zhang, Jindan Guo; Writing—Original Draft: Jiangning Liu; Writing—Review and Editing: Lihong Zhang, Jiangning Liu; Funding Acquisition: Jiangning Liu; Supervision: Jiangning Liu.

## CONFLICT OF INTEREST

The authors have no competing interests to declare.

## ETHICS STATEMENT

None.
